# Tianeptine Misuse, Dependence, and Clinical Management: Three Case Reports and Literature Review

**DOI:** 10.1155/crps/2981699

**Published:** 2026-04-20

**Authors:** Valeria A. Saldana, Paul H. Earley, David S. Silverstein, Yi-lang Tang

**Affiliations:** ^1^ Department of Psychiatry and Behavioral Sciences, Emory University School of Medicine, Atlanta, Georgia, USA, emory.edu; ^2^ Medical Director Emeritus, Georgia Professionals Health Program, Atlanta, Georgia, USA; ^3^ Longevity Services Inc., Spokane, Washington, USA; ^4^ Substance Abuse Treatment Program, Joseph Maxwell Cleland Atlanta VA Medical Center, Decatur, Georgia, USA

**Keywords:** dose escalation, misuse, substance use disorder due to another substance (tianeptine), tianeptine, withdrawal

## Abstract

Tianeptine, an antidepressant approved only in Europe, Asia, and Latin America, is available without regulation in the United States through online vendors and various retail locations. The rise in its misuse has raised public concerns, marked by dramatic dose escalation, rapid tolerance, toxicity, and withdrawal symptoms. Substance use disorder due to another substance (tianeptine) commonly presents with symptoms similar to opioid use disorder. Symptoms of intoxication include euphoria, respiratory depression, and CNS sedation. Withdrawal symptoms may include anxiety, agitation, and gastrointestinal distress. Acute intoxication management may include the use of naloxone and supportive measures. Withdrawal management should consider the use of buprenorphine as a first‐line treatment. Clonidine and other medications used for opioid withdrawal may also be beneficial. Cases with chronic use may require intensive interventions, including residential care, long‐term follow‐up, and close monitoring, as relapse rates are high. This report presents three cases of tianeptine misuse, detailing their presentation, withdrawal symptoms, and treatment, including pharmacological interventions and treatment setting. We further highlight the problems associated with misuse, the unique presentation of the disorder, treatment options for substance use disorder due to another substance (tianeptine), and the urgent need for regulatory oversight.

## 1. Introduction

Tianeptine, a tricyclic antidepressant widely available in parts of Europe, Asia, and Latin America, poses unique risks of misuse due to its opioid receptor activity. Though approved for major depressive disorder (MDD) in some countries, it remains unapproved by the U.S. Food and Drug Administration [1]. The therapeutic dose for depression typically ranges from 25 to 50 mg daily [2–4]. Tianeptine was initially believed to enhance serotonin reuptake; however, its primary mechanism involves glutamate modulation and μ‐opioid receptor agonism [5, 6], which has led to its misuse for euphoria and anxiolysis. Its rapid onset and short half‐life make it highly reinforcing, with users reporting a sense of well‐being and increased energy, which can lead to rapid dose escalation, repeated use, and dependence [2, 3]. Additionally, its glutamatergic modulation may enhance antidepressant effects and, thus reinforce its compulsive use [5]. Given its opioid receptor agonist properties, tianeptine misuse frequently mirrors opioid intoxication and withdrawal syndromes [2]. The U.S. Food and Drug Administration issued a warning to consumers in 2022 [7]; however, online and storefront vendors continue to openly market and sell this drug, contributing to its misuse.

The first cases of tianeptine misuse were reported in Europe, in patients who received prescribed tianeptine [8, 9], and its availability has since expanded. In the United States, tianeptine is frequently available at convenience stores, gas stations, vape shops, and online retailers. There are many names on the market, such as Tianaa, Zaza, Neptune’s Fix, Pegasus, and TD Red. Tianeptine has also been referred to as “gas station heroin” [4, 10]. A 2018 report by the Centers for Disease Control and Prevention highlighted a surge in tianeptine‐related exposure calls, emphasizing its emerging public health threat [3]. Poison control center data has shown a dramatic rise in tianeptine toxicity cases [11, 12], with over half of the cases requiring hospitalization, including intensive care unit admissions [11]. The symptoms of tianeptine toxicity are similar to opioid overdose, and severe cases often require hospitalization [11, 12]. Postmortem toxicological studies of two fatal cases found tianeptine concentrations of 2.0–8.4 mg/L, respectively [13]. While fatalities are rare, they can occur, particularly in cases of polysubstance use [13]. Early recognition and appropriate management are crucial to improving outcomes [8, 13, 14]. In this paper, we present three cases from our clinical encounters that illustrate the important symptoms, management, and treatment outcomes of patients with substance use disorder due to another substance (tianeptine). Our unique cases provide a detailed lens into individual motivations for tianeptine use, use patterns, treatment, and long‐term follow‐up information. Importantly, the cases presented were treated in a variety of clinical settings including an outpatient addiction clinic, an intensive outpatient program, and a residential rehabilitation facility, providing a broad view of treatment trajectories. Table 1 summarizes each of the cases clinical course and overall outcome.

**Table 1:** Summary of individual cases discussed in manuscript with demographics, tianeptine dose, intoxication and withdrawal symptoms, and clinical course.

Case	Demographics	Dose range	Intoxication symptoms	Withdrawal symptoms	Treatment	Outcome
Case A	Male in his 30s	Initially 75 mg per day, escalated to 50 g daily	Improvement in mood, anxiety, and energy	Anergia, anxiety, depression. With dose escalation, symptoms included rhinorrhea, cramping, vomiting, diarrhea, piloerection, sweating, and cold intolerance	Low‐dose buprenorphine during initial withdrawal symptoms. Setting was in an intensive outpatient program	Abstinent up until 3.5‐month follow‐up
Case B	Male in his late 30s	At peak use, consumed 12–15 g per day	Felt energetic	Anergia, depression, night sweats, worsening of pain, insomnia, and olfactory hallucinations	No medications were used for withdrawal or maintenance. Initially he was seen in an outpatient clinic and then was referred to residential treatment	Abstinent up until 4‐month follow‐up, remained in residential treatment program during this time
Case C	Male in his early 50s	Initially used 12.5 mg daily, escalated to up to 4–5 g daily	Improved energy and mood	Muscle aches, blurry vision, increased pain, and insomnia	Buprenorphine/naloxone, clonidine, and gabapentin as needed during initial withdrawal. He was initially seen in an outpatient clinic and was referred to an intensive outpatient program	Abstinent up to 3 months while in IOP. He relapsed again after completing program and was enrolled in a residential program. He remained abstinent with outpatient follow up and frequent drug monitoring as of the submission of this paper

## 2. Case Presentations

### 2.1. Case A

A man in his 30s with a history of severe opioid use disorder, developed substance use disorder due to another substance (tianeptine) after discovering it in the supplement industry. He endorsed interest in using tianeptine due to its reported anxiolytic effects. He purchased the powder of tianeptine online and the starting dose was approximately 75 mg per day. Initially, he reported feeling improvement in his mood, anxiety, and energy. Within weeks, he increased his dose to 150 mg multiple times throughout the day. Over a period of months, his dose rapidly increased to a self‐reported 50 g of tianeptine per day.

As his use progressed, he noted withdrawal symptoms approximately 3 h after his last dose. Symptoms initially presented as mood symptoms such as anergia, anxiety, and depressed mood. Symptoms escalated to rhinorrhea, cramping, vomiting, diarrhea, piloerection, sweating, and cold intolerance. He experienced intense cravings for tianeptine or other opioid substitutes, especially oxycodone. He would use buprenorphine (obtained on the gray market) to ameliorate opioid withdrawal symptoms when tianeptine was not available.

Regarding the criteria for opioid use disorders, he endorsed nine out of 12 DSM‐5 criteria [15], indicating a severe opioid use disorder. He noted a very rapid development of tolerance to the euphorigenic and anxiolytic properties. Importantly, he required rapid dose escalation to ward off withdrawal. This dose escalation accelerated much more rapidly than his previous hydrocodone and oxycodone misuse.

To treat his withdrawal from tianeptine, he was seen in an outpatient psychiatry clinic. No standardized opioid withdrawal scales were obtained. Although it was approximately 4 days after his last tianeptine use, he still required low‐dose buprenorphine for withdrawal management. Daily doses were 6, 6, 4, 4, 2, and 2 mg, respectively, with subsequent discontinuation. Gabapentin was used for his agitation and skin hypersensitivity, which he reported as moderately helpful. Shortly after discontinuation of tianeptine, laboratory studies showed mild elevations of aspartate aminotransferase (AST), alanine aminotransferase (ALT), alkaline phosphatase (ALP), and blood urea nitrogen (BUN), which were believed to be attributable to toxicity of high‐dose tianeptine. These returned to normal levels within 3 months. Problems with urinary retention continued for months after his last tianeptine use. Toxicological tests, including tianeptine and ethanol metabolites, obtained randomly at least twice per week, were continuously negative from admission to the point of our interviews 3.5 months later. These results align with expectations in the absence of tianeptine use within the preceding 3–4 days.

### 2.2. Case B

A man in his late 30s with a history of stimulant and gamma hydroxybutyrate (GHB) use disorders, developed substance use disorder due to another substance (tianeptine) after using it alongside other workout supplements. Prior to tianeptine use, he was sober and active in the recovery community for 15.5 years. In a manner like Case A, he started using tianeptine due to his interest in nutritional and workout supplements. He noted that he developed physiological dependence on tianeptine within several weeks. Initially, he felt sedated. Once he was dependent on tianeptine, he endorsed energizing effects. Missing doses would result in anergia and depression. At peak use, he consumed 12–15 g of tianeptine per day. Withdrawal symptoms included vomiting, night sweats, worsening of pain, insomnia, and olfactory hallucinations, and he received “detox” treatment at another facility. Objective withdrawal scores using COWS or CINA were not available. He met DSM‐5 criteria for severe substance use disorder due to another substance (tianeptine) [15] and was referred to a residential rehabilitation program. In the program, the patient received medications and therapies such as relapse‐prevention training, mood regulation skills, and cognitive behavioral therapy to build coping strategies and to help stabilize recovery. Several months after the last use, he continued to endorse intermittent periods of pressure in his head, tightness in his chest, and paresthesia of the extremities. He remained in the residential treatment program during the entire time up until our interview at the 4‐month mark. At that time, he achieved abstinence confirmed by comprehensive urine toxicological screens.

### 2.3. Case C

A male in his early 50s with a history of severe alcohol use disorder (in remission), experienced a depressive episode several years into sobriety. Seeking relief, he discovered tianeptine online and began using 12.5 mg daily, feeling energized and with improvement in his mood almost immediately. Overtime, he developed tolerance, escalating his dose up to 4–5 g daily, and experienced withdrawal symptoms including muscle aches, blurry vision, increased pain, and insomnia when attempting to quit. He attended an inpatient detox program and reported being treated with buprenorphine/naloxone, clonidine, and gabapentin as needed for withdrawal symptoms. Although external records were not available for review, this patient was evaluated by multiple authors of this paper in different clinical settings, and his report of use and prior management were consistent throughout the interviews. He met DSM‐5 criteria for severe substance use disorder due to another substance (tianeptine). Importantly, he endorsed intense cravings, tolerance, and withdrawal symptoms. He denied Criterion 3, which is defined as spending a great deal of time in activities to obtain, use, or recover from the substance. The patient was offered maintenance therapy with buprenorphine; however, he declined and stated that he believed he could maintain sobriety without medications. He was enrolled in an intensive outpatient program and achieved abstinence 3 months later, verified by frequent urine drug testing, including a special test for tianeptine. However, he relapsed after completing treatment and was again enrolled in another residential treatment program for 3 months. He remained abstinent with outpatient follow up and frequent drug monitoring as of the submission of this paper (October 2025).

## 3. Discussion

Tianeptine misuse may present with a range of physiological and behavioral symptoms consistent with DSM‐5 criteria for a substance use disorder, including compulsive use, tolerance, withdrawal, and significant distress or impairment in daily functioning [15, 16]. Springer and Cubała [17] reported that tianeptine dependence in Europe often begins with therapeutic use, which escalates to supratherapeutic doses (up to 4000 mg/day), driven by tolerance and cravings. Users in the United States often seek its euphoric effects or use it as a replacement for other substances. Through the social media platform Reddit, Smith et al. [18] found self‐reported drivers for interest in tianeptine use included its reported antidepressant effects and its impact on energy and quality of life, similar to our three cases [19]. While data from social media platforms should be interpreted with caution, the self‐reported motivations for tianeptine use expressed on this platform were consistent with our cases and those cited elsewhere in this paper.

### 3.1. Intoxication

#### 3.1.1. Opioid‐Like Effects

Acute tianeptine intoxication resembles opioid intoxication, particularly at high doses, with symptoms including euphoria, sedation, respiratory depression, and cognitive impairment [1–3]. Neurological symptoms include confusion, dizziness, and somnolence. Severe cases can lead to coma, and a case of tianeptine‐induced toxic leukoencephalopathy [20] was reported. Respiratory depression may require naloxone administration, though responses are inconsistent [11]. Cardiovascular effects include tachycardia, hypertension, and arrhythmias, while gastrointestinal symptoms such as nausea, vomiting, and constipation are also common. Psychiatric symptoms, including agitation, hallucinations, and paranoia, have also been reported [3]. Severe cases may require ICU admission and mechanical ventilation.

### 3.2. Withdrawal

#### 3.2.1. Opioid‐Like and Unique Symptoms

Tianeptine withdrawal is often severe, resembling opioid withdrawal but with unique features [2, 17]. Common opioid withdrawal symptoms include anxiety, agitation, insomnia, myalgia, piloerection, rhinorrhea, yawning, nausea, vomiting, diarrhea, tachycardia, and hypertension. However, distinct symptoms such as olfactory hallucinations, urinary retention, constipation (not diarrhea), and sensory hypersensitivity have been documented and these symptoms were seen throughout our three cases [16, 17, 21]. Notably, Case B experienced diarrhea initially during withdrawal. Tianeptine withdrawal symptoms typically begin within hours of the last dose due to tianeptine’s short half‐life, peaking within 24–48 h [21]. Some reports suggest physical withdrawal symptoms typically last one to 2 weeks. Case A also reported prolonged urinary retention for several months after his last use of tianeptine. Cravings may extend for quite some time and can be unrelenting and often contribute to relapse [22].

### 3.3. Assessment and Diagnosis

Recognizing substance use disorder due to another substance (tianeptine) requires clinical awareness and careful history‐taking. Importantly, standard toxicology screens do not include tianeptine and its metabolites [4]. Unlike traditional opioids, tianeptine undergoes minimal hepatic metabolism and is primarily excreted unchanged in urine, reducing hepatotoxicity risks but complicating toxicological detection. Tianeptine assays are completed at a reference lab in most cases, and results often do not return for days to weeks. Due to regulatory gaps and easy availability, tianeptine misuse is often overlooked or misdiagnosed. Physicians should inquire about non‐prescription substance use and consider specialized toxicology tests to aid in diagnosis. Clinicians should be familiar with the common street and “sold as” names to ensure an accurate diagnosis. Tianeptine misuse should be differentiated from opioid use disorder, benzodiazepine withdrawal, and stimulant‐induced anxiety. Withdrawal commonly features constipation instead of diarrhea.

### 3.4. Treatment

Common presentations of substance use disorder due to another substance (tianeptine) include intoxication and withdrawal, which require immediate and specific interventions. However, long‐term recovery and relapse prevention are equally crucial. Effective treatment plans should address both the acute phases of intoxication and withdrawal, as well as provide ongoing support and strategies to maintain sobriety and prevent relapse. The cases described in this report were managed across diverse clinical settings, including an outpatient addiction clinic, an intensive outpatient program, and a residential rehabilitation program. Some individuals (such as Case C) received a combination of outpatient and residential treatment.

### 3.5. Acute Intoxication Management

Acute tianeptine intoxication poses a serious risk due to its opioid‐like effects, such as respiratory depression and central nervous system sedation. Both intranasal or intravenous naloxone should be prioritized and is the primary pharmacological intervention. Higher doses (up to 10 mg) may be required due to tianeptine’s high receptor‐binding affinity [11, 23], especially in cases involving extraordinarily high doses of tianeptine. Naloxone’s effectiveness can be variable. Severe cases, or those with concurrent overdose of other medications (such as benzodiazepines), may require mechanical ventilation and intensive supportive care [24, 25]. Fluid resuscitation and electrolyte correction are recommended to stabilize cardiovascular function. Comprehensive toxicology screening is crucial to guide appropriate intervention due to frequent co‐ingestion of substances like benzodiazepines and alcohol.

### 3.6. Withdrawal Management

Tianeptine withdrawal resembles opioid withdrawal and often requires a combination of pharmacologic and supportive treatments. As standardized tianeptine use and withdrawal protocols are unavailable, several studies and clinical cases follow opioid use disorder guidelines. Buprenorphine, a partial μ‐opioid receptor agonist, is the first‐line therapy for alleviating withdrawal symptoms, managing cravings, and reducing relapses [2, 22, 26]. Sublingual administration is generally preferred, and rapid symptom relief has been reported with early buprenorphine initiation similar to opioid use disorder (4–16 mg/day), while microdosing is also an option [27]. Methadone has also been reported as a treatment option for tianeptine withdrawal management [28]. If using methadone for maintenance, the dosing protocol can also follow established guidelines for opioid use disorder. Clonidine, an alpha‐2 adrenergic agonist, is effective for managing autonomic withdrawal symptoms and is commonly used with loperamide for diarrhea and NSAIDs for myalgias [26, 27]. Additional adjunctive medications include gabapentin for neuropathic pain and restlessness, trazodone to address insomnia and depressive symptoms, and benzodiazepines for anxiety and insomnia [2, 16, 21].

### 3.7. Long‐Term Treatment and Relapse Prevention

For long‐term management, maintenance medications should be considered. Buprenorphine/naloxone maintenance therapy is recommended for individuals with severe substance use disorder due to another substance (tianeptine) or concurrent opioid use disorder [16, 17, 26]. Naltrexone (especially the injectable), an opioid antagonist, may be used in abstinent patients to prevent relapses, though adherence remains a challenge. Methadone maintenance has also been used in some cases [28, 29]. In the meantime, treating preexisting or comorbid mental disorders (such as depression) may reduce the risk of relapse.

Psychosocial interventions are also critical. Specialty care (addiction treatment) is often required, including intensive outpatient programs or residential rehabilitation programs. Many therapeutic modalities can be used, such as cognitive behavioral therapy to help recognize and modify maladaptive thoughts and behaviors related to substance use, contingency management to reinforce positive behaviors with tangible rewards, and relapse prevention skills training to maintain abstinence. Individuals early in remission should engage in ongoing therapy and be coached to avoid online shopping and purchasing gasoline at locations that sell the drug. Additionally, peer support programs like Narcotics Anonymous (NA) or SMART Recovery offer a sense of community and accountability, which improve long‐term outcomes [30].

### 3.8. Growing Public Concern

While data for substance use disorder due to another substance (tianeptine) remains limited, more international reports suggest a growing trend. In countries where tianeptine is prescribed, such as France and Turkey, misuse is noted often among individuals with a history of substance use disorders [22]. Online availability has further fueled its misuse, allowing easy access without a prescription.

In the United States, cases of tianeptine exposure have risen sharply in the past 10 years. Between 2000 and 2013, only 11 cases were reported to the National Poison Data System, but from 2014 to 2017, the number surged to 218, with the highest concentration in the Southern United States [3]. Most affected individuals are men aged 21–40 years with a history of substance use disorder. Similarly, a New Jersey report (2023) documented 17 cases of severe toxicity over 6 months, with symptoms including altered mental status, seizures, cardiac arrest, and prolonged QT intervals. Many required ICU admission and mechanical ventilation, and had coexposures to other substances like alprazolam, kratom, and gabapentin. Mass spectrometry performed of samples from two cases found variable product composition, including different synthetic cannabinoid receptor agonists [31]. Earlier data from New York Poison Control Centers also showed concerning trends [32]. In Tennessee, 50 emergency department visits, six fatal overdoses, and 19 drug seizures related to tianeptine use were recorded from 2021 to 2023 [12].

Springer and Cubała [17] reported similar cases across Europe, particularly in France, Turkey, and Poland. Their review of 18 case reports found that users frequently escalated doses to supratherapeutic levels, sometimes exceeding 4000 mg/day. A history of substance use disorders was a common factor among those who developed dependence.

Regulatory gaps continue to enable widespread misuse. Tianeptine remains unscheduled in many jurisdictions, leading to easy accessibility via online markets and gas stations [1, 7]. Due to being an unregulated product, tianeptine products may differ in composition and may contain other synthetic substances that can potentiate adverse effects. The labels of tianeptine products obtained via the gray market may not be reliable and may not reflect the true tianeptine content in their products. Healthcare providers’ awareness of the dangers of tianeptine remains low. Just this year, the FDA issued another public warning for healthcare professionals regarding tianeptine use trends and identification, adverse events, and resources available for patients [15]. Its unregulated status complicates prevention and treatment efforts.

Our cases illustrate the unique presentation of substance use disorder due to another substance (tianeptine), including adverse effects associated with use and approaches to treatment management. A key limitation is the lack of patient diversity, as all three cases involved young to middle‐aged males in different treatment settings. Additionally, these cases were collected over several years, which limited our ability to obtain standardized objective withdrawal scores, clinician, and patient perspectives. Despite these limitations, the cases offer several strengths: they provide detailed accounts of dose escalation, individual perspectives on intoxication effects driving continued use, withdrawal symptoms, individualized treatments used, and long‐term treatment outcomes. Further research and case studies should aim to include a broader demographic representation, a more detailed assessment of substance use history, and explore the underlying motivations for tianeptine use.

## 4. Conclusions

Tianeptine misuse is emerging as a public health concern. The rapid tolerance development and dose escalation are hallmark presentations that drive use. The clinical presentations associated with tianeptine misuse align with DSM‐5 criteria for a substance use disorder. Patients often present with opioid‐like intoxication and withdrawal symptoms that can result in serious adverse outcomes and hospitalization. The three cases described in our paper provide detailed narratives about substance use history, the acquisition of tianeptine, motivations for use, patterns of tolerance and dose escalation, medication management, and the clinical settings in which treatment is provided. These cases underscore the clinical implications of tianeptine’s increasing availability, poor regulation, and potential for misuse, highlighting the urgent need for greater awareness among healthcare providers and federal regulators. Further research is required to establish standardized treatment protocols for tianeptine withdrawal, toxicity, and relapse prevention.

## Author Contributions

**Valeria A. Saldana and Yi-lang Tang:** conceptualization (lead), clinical interviews (lead), manuscript preparation (lead). **Paul H. Earley:** clinical interviews (lead), manuscript preparation (lead). **David S. Silverstein:** toxicological test development (lead), manuscript preparation (supporting).

## Funding

The authors have nothing to report.

## Disclosure

The manuscript has not been posted on a preprint server. Yi‐lang Tang’s place of employment had no impact on the outcome of our case series. The views expressed in this paper are those of the authors and do not necessarily reflect the position or policy of the Department of Veterans Affairs or the United States government.

## Consent

Written informed consents for publication were obtained from all cases reported. All efforts have been made to protect patients’ privacy and confidentiality.

## Conflicts of Interest

Yi‐lang Tang is a federal employee at the Joseph Maxwell Cleland Atlanta VA Medical Center. The other authors declare no conflicts of interest.

## Data Availability

Data sharing is not applicable to this article as no new data were created or analyzed in this study.
